# Specific requirements for automation of immunoassays in 1990

**DOI:** 10.1155/S1463924691000123

**Published:** 1991

**Authors:** R. Haeckel

**Affiliations:** Institut für Laboratoriumsmedizin Zentrallabor Zentralkrankenhaus St-Jürgen Strasse Bremen Germany


Journal of Automatic Chemistry, Vol. 13, No. 2 (March–April 1991), pp. 53–56

### Specific requirements for automation of immunoassays in 1990

**R. Haeckel**
*Institut für Laboratoriumsmedizin, Zentrallabor, Zentralkrankenhaus St-Jürgen Strasse, Bremen, Germany*

### Introduction

Immunoassays are still gaining importance in the central laboratories of large hospitals (see table 1). This trend means that automated techniques are becoming more desirable for immunoassays and many manufacturers are developing new analytical systems. Therefore, it is valuable to examine the technical and analytical requirements for these devices.

### Technical requirements

More general requirements refer to automation independently of the type of analytical system or of the tests performed: for example free positioning of samples, several barcodes for sample identification, bidirectional communication with central data processing system, small void volume (50 μl), detection of sample exhaustion, speed, reasonable size. This list is not comprehensive and contains only the most common claims, because these claims are not specific to immunoassays, they will not be discussed in any more detail in this paper.

Among specific claims for immunoassays, 4 major claims should be mentioned: selective multitesting, full automation, completion of panels and high throughput rate.

Since several years many laboratories have claimed selective multitesting as it is now satisfactorily solved in classical clinical chemistry. This need is now recognized by industry and many companies are developing such analytical systems for immunoassays. Selective multitesting should not be confused with the term random access which should be replaced by the term selective access [1].

*Table 1. Number of the 10 most often requested immunoassays during January in the central laboratory of a 1500 bed hospital.*

| | 1988 | 1989 | 1990 | 1991 |
|---|---|---|---|---|
| TSH | 307 | 301 | 356 | 385 |
| $T_4$ | 308 | 284 | 371 | 247 |
| $T_3$ | 233 | 232 | 287 | 268 |
| Ferritin | 176 | 200 | 232 | 242 |
| $\beta_2$-Mikroglobulin | 8 | 113 | 171 | 238 |
| CEA | 359 | 252 | 245 | 237 |
| PSA | 54 | 171 | 201 | 221 |
| CA 19-9 | 158 | 121 | 125 | 151 |
| Cortisol | 126 | 113 | 97 | 144 |
| Freies Östriol | 151 | 148 | 124 | 138 |

Very often new immunoassay systems are introduced with a test panel based on the most commonly used tests. In the central laboratory of our hospital, about 70 various immunoassays are performed. The first 10 of the most frequently applied tests are listed on table 1. This list most probably differs from laboratory to laboratory and contains a very heterogenous group. Most laboratories would not like to buy a new system which could only deal with these tests and rather prefer analytical systems which perform complete panels of groups which are referred to specific disease entities such as complete thyroid panel or hepatitis panel, instead of a mixture of most commonly used tests (table 2).

The throughput rate of automated immunoassays should be at least 100 tests (results) per hour and short incubation times of less than one hour if the system is designed for larger laboratories.

### Analytical requirements

### *Principles*

All immunoassays are based on the reaction of an antigen with a more or less specific binding protein, which in most cases is an antibody (see figure 1). The design of this reaction needs consideration of several other auxiliary reactions, all of which can cause problems. This is demonstrated in figure 1 for a solid phase technique. This reaction can be directly detected by turbidity measurements, or indirectly detected by including a 'tracer'. Tracers have been radioisotopes for nearly three decades. There is now a clear trend to replace these tracers by non-isotopes for two principal reasons: to avoid radioactivity, and to improve the chances of automation. Several solutions are now commercially available, which provide sufficient analytical realibility (see table 3).

Three detection principles are presently used: absorbance, fluorescence and luminescence. Luminescence is the newest solution. Although luminescence was first described more than 300 years ago, it has been shown only very recently by highly sensitive photomultipliers that luminescence is a universal property of all organic compounds that can be oxidized. This luminescence is mostly minimal and not suitable for analytical purposes. However, during the last decades about 100 substances have been detected which have sufficient light emission to be used by the commonly applied detection systems. The most suitable substances are listed in table 3.

There is already a confusing variety of technologies. They can be classified by three principles:

(1) Type of label or tracer.

0142–0453/91 $3.00 © 1991 Taylor & Francis Ltd.

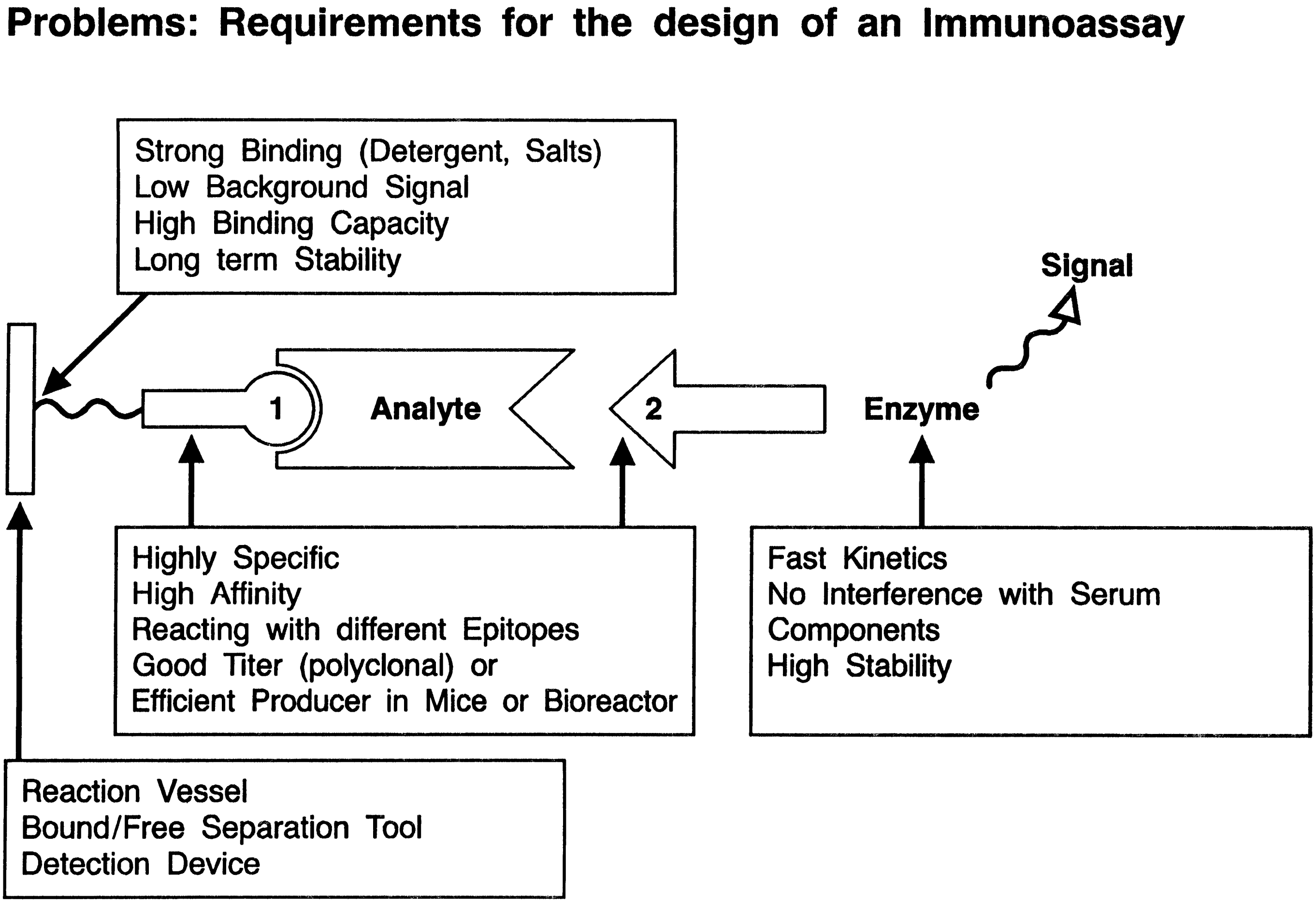

*Figure 1. Requirements for the design of an immunoassay (provided by Dr Kawaletz, Boehringer Mannheim Corp.). The numbers represent binding proteins (e.g. antibodies).*

(2) Inclusion of a separation step (homogenous/ heterogenous test).

(3) Competitive principle or extraction-saturation.

Masseyef has proposed a three-dimensional scheme [2], which, however, is only valid for a rough orientation.

*Acceptability criteria*

*Precision*

In classical clinical chemistry, many procedures have a linear relationship between standard deviation and analyte concentration. With most immunoassays the precision profile is more or less like a parabel (figure 2). Therefore, precision limits must be defined for various intervals of the whole analytical range. This will be demonstrated with thyrotropin (TSH) as an example (figure 3): the reference range covers one order of magnitude (0·3–0·4 mU/l), the range below 0·1 indicates hyperthyroidism; the range above 10·0, hypothyroidism with high certainty. Which precision is required in the three parts of the analytical range? Another question is: what interval between A and B should be chosen, or can be omitted?

It is now generally accepted that analytical imprecision goals can be based on the biological variability. However, several proposals have been issued concerning the acceptable fraction of the analytical variability (table 4). According to the first proposal in table 4, the analytical imprecision for TSH should be equal or smaller than 8·1 [3]. This goal is usually considered to be independent of sex and age, and to be constant in the reference range. When the 'ultrasensitive TSH' tests were developed, the discussion on analytical goals in the lower part of the analytical range was renewed.

A special case is the imprecision of the blank which is used as a measure of the detection limit. A survey of definitions has recently been published [5]. It is generally considered to be the minimally detectable quantity.

*Table 2. Immunoassay panels.*

| | |
|---|---|
| 1. | Thyroid: $T_4$, $T_3$, T-uptake, TSH, FT4 |
| 2. | Anemia: $B_{12}$, folate, ferritin |
| 3. | Fertility: hCG, FSH, LH, prolactin, estradiol, progesterone, testosterone |
| 4. | Pregnancy: hCG, AFP, HPL, estriol |
| 5. | Other hormones: cortisol, STH, PTH |
| 6. | Hepatitis: HBsAg, HBeAg, anti-HBs, anti-HBe, anti-HBc, anti-HBcIgM, anti-HAV, anti-HAVIgM, anti-HCV |
| 7. | Other infectious diseases: chlamydia, HIV |
| 8. | Tumour markers: CEA, AFP, PSA, Ca 19-9, Ca 12-5 |

*Table 3. Non-isotopic immunoassays.*

| Method | Label | Analytical system (distributor) |
|---|---|---|
| *Absorbance* | | |
| ELISA | ABTS/POD | ES 600 (BMC) |
| EIA | phenolphtalein/AP | SR 1 (Serono) |
| EMIT | NADH/G6PDH | Spektrometer (Syva) |
| EASIA | TMB/POD | Vmax (Medgenix) |
| *Fluorescence* | | |
| FPIA (polarization) | fluorescein | TDX (Abbott) |
| MEIA (microbeads) | umbelliferon/AP | IMX (Abbott) |
| TRFIA (time resolved) | Eu $(\beta NTA)_3$ | Delfia (Pharmacia) |
| FEIA (radial partition) | umbelliferon/AP | Stratus (Baxter) |
| FETI | fluorescein | Advance (Syva) |
| *Luminescence* | | |
| LEIA (enhanced) | luminol/POD | Amerlite (Amersham) |
| CLIA | acridiniumester | Magic-Lite (Ciba Corning) |
| SPALT (solid phase) | acridiniumester | Berilux (Behring) |
| ILMA | ABEI/AP, POD | Liamat (Byk-Sangtec) |

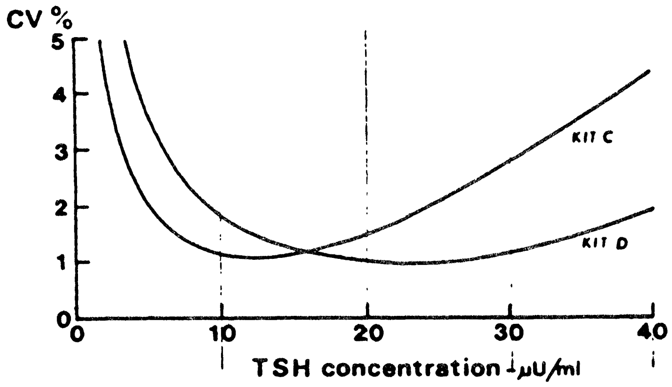

*Figure 2. Typical relationship between the coefficient of variation and the analyte concentration of an immunoassay.*

Again, several proposals exist for the measurement of imprecision of the blank (table 5). Ekins has suggested calculating imprecision from duplicates of patients' samples taken from the whole analytical range, to calculate standard deviations from several intervals of the analytical range and then to extrapolate to the theoretical standard deviation for an analyte free sample, that means to a real blank value [4]; Ekins' procedure may imitate 'the real sample conditions' more realistic than aqueous standard solutions with an artificial matrix [5].

If the detection limit is defined, and if it is below the lower end of the reference range, the following claims can be recommended for the determination of the TSH activity:

(1) 1% criterion for detection limit ($D_L$ = 0·05 U/l).

(2) $Cv_A$ at lower limit of the reference range ≤8%.

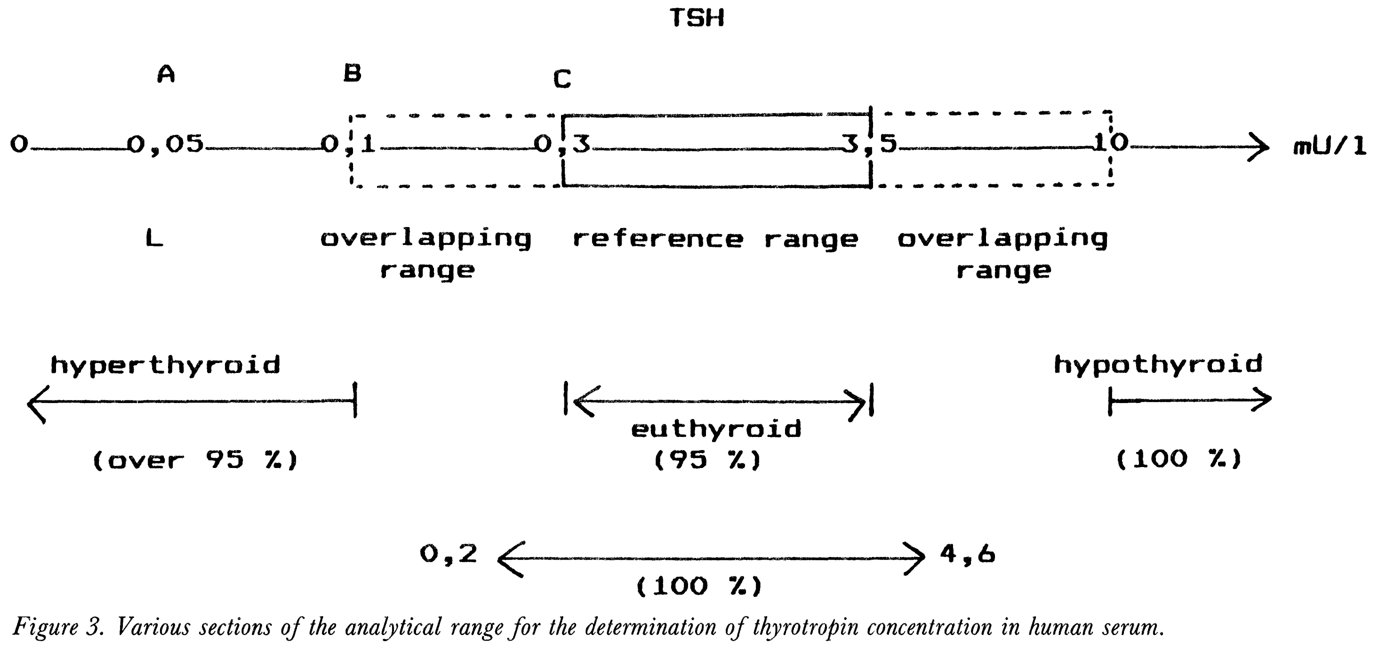

*Figure 3. Various sections of the analytical range for the determination of thyrotropin concentration in human serum.*

*Table 4. Acceptable limits of imprecision, CV = coefficient of variation, A = analytical, B = biological variability.*

1. Cotlove, Harris, Fraser [7–9]
   (intraindividual variability)
   $CV_A \leq 1/2\ CV_B$
2. Stamm [6]
   (interindividual variability)
   $CV_A \leq 1/3\ CV_B \sim 1/12$ reference range
3. NCCLS [10]
   (intraindividual variability)
   $CV_A \leq 1/4\ CV_B$

(3) The calibrator with the lowest concentration should be between $D_L$ and the lower limit of the reference range.

Analogue considerations can be made for other immunoassays.

*Accuracy*

The accuracy of many immunoassays, especially for hormone analyses, has two major problems which still require substantial international effort: the calibration of the assays and the specificity of the antibodies.

*Standardization of calibrators:* In the case of peptides and protein antigens, calibration of the various procedures depend on the purity and definition of the calibrators. In most cases it is not yet possible to produce calibrators with the purity required at a reasonable cost. Therefore, the only possible way for this moment, is to trace back calibration to calibrators with internationally accepted concensus values. Such international reference materials are usually produced by or under the auspices of WHO, or similar organizations.

*Specificity of antibodies:* Much progress has been achieved by the introduction of monoclonal antibodies. A next important step should be epitope mapping to define antibody specificity and comparison with bioassays to prove the biorelevance of the epitope detected by the monoclonal antibodies. The specificity of antibodies for particular antigen epitopes is still relatively unknown.

*Table 5. Procedures for determining the detection limit [5].*

| | |
|---|---|
| 1. | Blank sample methods |
| 1.1 | One series |
| 1.2 | Multiple blank duplicates |
| 2. | Precision profile methods |
| 2.1 | Using calibrators |
| 2.2 | Using unknown samples |

In the case of proteins, the antigen characteristics depend on the physical-chemical structure relationship. That means: the availability of antigen determinants are variable. Denaturation and fragmentation can lead to loss of antigen determinants or even to new determinants which are called 'additive epitopes'.

*Acceptability criteria for inaccuracy:* Criteria for the acceptability of inaccuracy are usually based on imprecision criteria, for example [5]:

$$\frac{x_i - x_R}{x_R} 100 \leq 3\ CV_A$$

$x_R$ = reference method value, $x_i$ = value measured and $CV_A$ = coefficient of variation (analytical goal calculated from biological variability).

For TSH this would mean that inaccuracy should be less than 24%. With a two standard deviation range it would be 16%.

### Conclusion

In conclusion, analytical performance criteria should be established for each test, as demonstrated for TSH, and these goals must also be reached by automated systems.

### References

1. Haeckel, R., Burtis, C. A. and Geary, T. D., *Clinical Chemistry,* **34** (1988), 1520.
2. Masseyef, R., Pour une sémantique de l'immunoanalyse. In: *Communication, 7es Journées Nationales de la SFBC,* Deauville, 28 juin (1989).
3. Browning, M. C. K., *Annals of Clinical Biochemistry,* **26** (1989), 1–12.
4. Ekins, R. P., In W. M. Hunter and J. E. T. Corrie (Eds), *Immunoassays for Clinical Chemistry* (Churchill Livingstone, Edinburgh, 1983), 76–105.
5. Haeckel, R., Colic, D. and Baud, M., *Journal of Clinical Chemistry and Clinical Biochemistry* (in press).
6. Stamm, D., *Dt. Ärzteblatt,* **35** (1988), B517–519.
7. Cotlove, E., Harris, E. K. and Williams, G. Z., *Clinical Chemistry,* **16** (1970), 1028–1032.
8. Harris, E. K., *American Journal of Clinical Pathology,* **72** (1979), 374–382.
9. Fraser, C. G., *Arch. Pathol. Lab. Med.,* **112** (1988), 404–409.
10. O'Sullivan, M. B., Bessman, D., Bull, B. S., Groner, W., Klee, G. G., Koepke, J. A., Richardson-Jones, A., Simson, E., van Assendelft, O. W. and von Behrens, W., Performance goals for the internal quality control of multichannel hematology analyzers. *NCCLS,* **9**, No. 9, ISSN 0273-3088 (1989).

